# Genomic Epidemiology of *Vibrio cholerae* O139, Zhejiang Province, China, 1994–2018

**DOI:** 10.3201/eid2811.212066

**Published:** 2022-11

**Authors:** Yun Luo, Julian Ye, Michael Payne, Dalong Hu, Jianmin Jiang, Ruiting Lan

**Affiliations:** Zhejiang Provincial Center for Disease Control and Prevention, Hangzhou, China (Y. Luo, J. Ye, J. Jiang);; University of New South Wales, Sydney, New South Wales, Australia (Y. Luo, M. Payne, D. Hu, R. Lan);; Key Laboratory of Vaccine, Hangzhou (J. Jiang)

**Keywords:** cholera, *Vibrio cholerae*, O139, genomic, epidemiology, Zhejiang, China, bacteria, bacterial infections, waterborne diseases, antimicrobial resistance

## Abstract

Cholera caused by *Vibrio cholerae* O139 was first reported in Bangladesh and India in 1992. To determine the genomic epidemiology and origins of O139 in China, we sequenced 104 O139 isolates collected from Zhejiang Province, China, during 1994–2018 and compared them with 57 O139 genomes from other countries in Asia. Most Zhejiang isolates fell into 3 clusters (C1–C3), which probably originated in India (C1) and Thailand (C2 and C3) during the early 1990s. Different clusters harbored different antimicrobial resistance genes and IncA/C plasmids. The integrative and conjugative elements carried by Zhejiang isolates were of a new type, differing from ICEVchInd4 and SXT^MO10^ by single-nucleotide polymorphisms and presence of genes. Quinolone resistance–conferring mutations S85L in *parC* and S83I in *gyrA* occurred in 71.2% of the Zhejiang isolates. The *ctxB* copy number differed among the 3 clusters. Our findings provided new insights for prevention and control of O139 cholera .

Cholera is an acute watery diarrheal disease that has caused 7 global pandemics since 1817. The current, ongoing seventh pandemic started in 1961 and continues today (*1*). The causative agent of cholera is *Vibrio cholerae*; serogroups O1 and O139 cause epidemic- and pandemic-level disease. Serogroup O139 first caused an outbreak in Bangladesh and India in 1992 (*2*,*3*). However, the epidemic O139 clone was later found to be a derivative of a seventh pandemic O1 strain, having had its O1 gene cluster replaced with an O139 O antigen gene cluster (*4*) and therefore genetically belonging to the seventh pandemic clone and sharing the same sequence type (*5*).

*V. cholerae* O139 spread to China and was reported in Xinjiang Uygur Autonomous Region (*6*) and Guangdong Province (*7*) in 1993 and in Jiangxi Province and the cities of Beijing and Shanghai in 1994 (*8*). Most studies on O139 in China have focused on virulence and resistance-gene profiles, cholera toxin (CTX) types (*7*,*8*), and plasmid carriage (*9*). The genomic epidemiology of O139 in China and the phylogenetic relationship of isolates from China to isolates from other countries in Asia are still unknown. A study of 9 O139 isolates suggested that O139 reached China soon after outbreaks in India in early 1990s and became dominant a few years later (*10*).

Antimicrobial therapy (in addition to rehydration therapy) plays a vital role in the management of cholera patients (*11*). In a previous study of 340 O139 isolates collect in China during 1993–2009, resistances to streptomycin, trimethoprim/sulfamethoxazole, and polymyxin B were found in isolates from early years (*12*). IncA/C conjugative plasmids can effectively mobilize genes associated with resistance to different classes of antibiotics, including β-lactams, aminoglycosides, chloramphenicol, folate-pathway inhibitors, quinolones, and tetracycline (*13*). IncA/C plasmids are widely present in Enterobacterales but not common in *V. cholerae* populations (*14*), although they have been found in the seventh pandemic *V. cholerae* lineage (*11*).

In this study, we sequenced the genomes of 104 *V. cholerae* O139 isolates collected from Zhejiang Province, China, during 1994–2018. Comparative genomic and phylogenetic analyses revealed the genetic characteristics of *V. cholerae* O139 isolates in Zhejiang and their evolutionary relationships to isolates from countries in Asia. We also analyzed the virulence and antimicrobial resistance (AMR) gene profiles and the distribution of IncA/C plasmids to elucidate the evolution of virulence and AMR.

## Methods

### Isolates

We recovered 104 *V. cholerae* O139 isolates collected during 1994–2018 from the Zhejiang Provincial Center for Disease Control and Prevention (Zhejiang CDC). We downloaded 57 public *V. cholerae* genomes from countries in Asia (Appendix 1 Table 1) and 133 publicly available *V. cholerae* genomes from China from the European Nucleotide Archive database (https://www.ebi.ac.uk/ena) and identified them by searching for the O139 O-antigen–specific *wbf* gene using BLASTN version 2.9.0 (*15*).

### Genome Sequencing

We performed whole-genome sequencing by using the Illumina Hiseq X-ten sequencing platform with TruePrepTM DNA Library Prep Kit version 2 and 150-bp paired-end sequencing (Illumina, https://www.illumina.com). We checked all input read sets for contamination by using kraken2 with a threshold of 10% for non–*V. cholerae* reads (*16*). We submitted genome sequences obtained in this study as raw reads under the National Center for Biotechnology Information’s Sequence Read Archive database (Bioproject no. PRJNA643344).

### Single-Nucleotide polymorphism Calling and Phylogenetic Analyses

We identified single-nucleotide polymorphisms (SNPs) by using a section of the SaRTree (*17*) pipeline. We removed Superintegron sequences on the small chromosome and all recombinant SNPs. The reference genome sequence (GenBank accession no. GCF_900324445.1) was from Bangladesh strain 4295STDY6534216, isolated in 2014 (*18*). We allocated SNPs to each branch of the tree by using the SaRTree pipeline (*17*). We performed phylogenetic analysis by constructing a maximum-likelihood tree using IQ-Tree version 2.0.4 (*19*) under default parameters (transversion model with AG = CT and empirical base frequencies) with 1,000 bootstrap replicates.

### Antimicrobial-Resistance and Virulence Genes

For all genomes, we predicted AMR genes by using ABRicate (https://github.com/tseemann/abricate) with the AMRFinderPlus gene database (*20*), plasmids by using PlasmidFinder (*21*), and virulence genes by using a customized database of 67 virulence genes (Appendix 1 Table 2). We applied a cutoff of percentage nucleotide identity at 80% for virulence genes and plasmids and at 60% for resistance genes. We used k-mer alignment (*22*) to map raw reads against all these genes. As criteria for gene presence, we used a combination of minimum identity and coverage thresholds from ABRicate or the ratio of the gene depth to the average depth of housekeeping genes >20% from KMA. We used CNVnator version 0.4.1 (*23*) with default settings and a bin size of 100 bp to calculate copy numbers of *ctxB* genes.

## Results

### Whole-Genome Sequencing of *V. cholerae* O139 Isolates from Zhejiang Province

**Figure 1:**
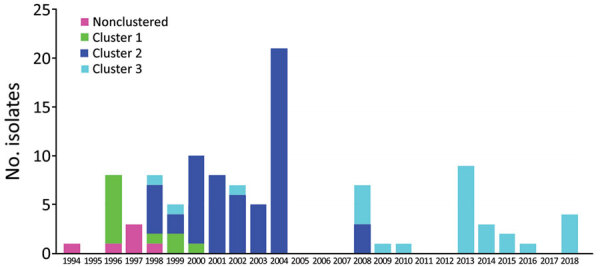
Distribution of *Vibrio cholerae* O139 isolates, by clusters and year of isolation, Zhejiang Province, China, 1994–2018. Bar sections represent isolate numbers in different clusters in each year (Appendix 1 Table 4; Appendix 2 Figure 2).

We recovered and sequenced 104 *V. cholerae* O139 isolates collected during 1994 to 2018 by Zhejiang CDC (Figure 1; Appendix 1 Table 1). For comparison with other isolates from China, we included 9 published isolates from Shanghai (*10*). We used another 133 publicly available O139 genomes from China without metadata only to infer phylogenetic relationships with Zhejiang isolates. For international comparison, we included 48 publicly available O139 genomes from India (*19*), Bangladesh (*19*), and Thailand (*10*). The earliest isolates were collected in 1983 in Bangladesh; other isolates were collected during 1992–2014 (Appendix 2 Figure 1).

### Phylogenetic Analysis of O139 Isolates from China and Worldwide

**Figure 2:**
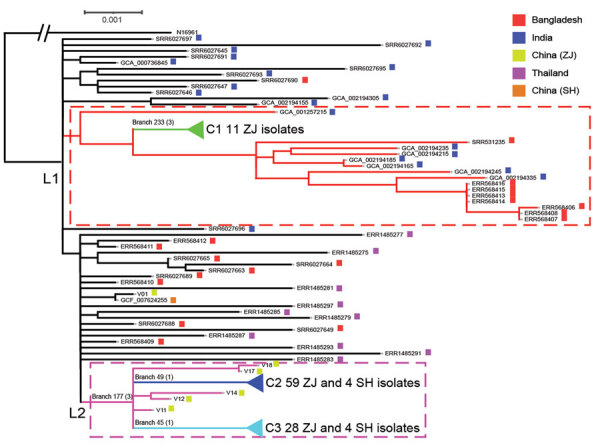
Maximum-likelihood phylogenetic tree of 161 *Vibrio cholerae* O139 (sequence type 69) isolates from Zhejiang Province, China, 1994–2018, and isolates from outside of China. The tree was rooted using the seventh pandemic O1 strain N16961 as an outgroup. Lineage 1 (L1) and lineage 2 (L2) are demarcated with red dashed lines and pink dashed-line boxes. The 3 clusters (C1, C2, and C3) are collapsed to reduce figure size (Appendix 2 Figure 2). Key branches are marked with a branch number followed in brackets by the number of single nucleotide polymorphisms that supported the branch. The colored solid squares at the end of isolate names indicate the location of isolation of the isolates. GenBank accession numbers were used as isolate names for O139 isolates not from Zhejiang Province. SH, Shanghai; ZJ, Zhejiang.

We identified 629 SNPs from our 104 Zhejiang O139 isolates, 9 Shanghai isolates, and 48 isolates from other countries; 501 SNPs were on chromosome I and 128 SNPs were on chromosome II. We constructed a phylogenetic tree of the 161 isolates, using N16961 as the outgroup (Figure 2; Appendix 2 Figure 2). We further identified branch-supporting SNPs (Appendix 1 Table 3; Appendix 2 Figure 3). The tree can be divided into 2 distinctive linages, defined as lineages 1 (L1) and 2 (L2). L1 contained 11 isolates from this study and 15 isolates from Bangladesh and India. L2 contained 92 isolates from this study and 8 Shanghai isolates.

The isolates from China grouped together as 3 clusters, which were each supported by unique SNPs (Appendix 2 Figures 2, 3). Cluster 1 (C1) consisted of 11 Zhejiang isolates collected during 1996–2000 supported by 3 SNPs on branch 233. Cluster 2 (C2) consisted of 59 isolates from this study and 4 Shanghai isolates collected during 1998–2008. C2 was supported by 1 SNP on branch 49. Cluster 3 (C3) consisted of 28 isolates from this study and 4 isolates from Shanghai collected during 1998–2018. C3 was supported by 1 SNP (branch 45). C1 was located within L1, whereas C2 and C3 were located within L2. The closest ancestral isolate of C1 was an isolate from India. C2 and C3 grouped together as L2, and all isolates in L2 originated in China. The L2 node was supported by 3 SNPs (branch 177), and the closest ancestral isolate of L2 was from Thailand.

Six isolates from Zhejiang fell outside of C2 and C3 and were referred to as outliers. These 6 isolates were obtained from patients in the 1990s. One isolate (V01) was isolated in 1994 from the first clinical case-patient in Zhejiang but shared a common ancestor with 1 isolate from Shanghai (isolated in 1994) and was sibling to L2. 

We also constructed a phylogenetic tree of 349 genomes that included the 133 isolates from China without metadata (Appendix 2 Figure 4). The 2 lineages (L1 and L2) and 3 clusters were preserved on this tree. Most isolates from China fell into C2 (92/133 [69.2%]), 9 isolates fell into C3, and only 2 isolates fell into C1.

### Genetic Elements and Virulence Genes

We searched the 104 genomes from this study by using ABRicate with our custom database of 67 known *V. cholerae* virulence genes. We further confirmed by reads mapping any genomes with a negative result for any of these virulence genes. The presence of a gene was a combined result of ABRicate searches of assembled genomes and reads mapping. Four core CTX phage genes (*ace*, *zot*, *ctxA*, and *ctxB*) and the repeat sequences were all present in 94 genomes. Three genomes (V31, V32, and V33) were all negative for these genes. The repeat sequences were not well assembled and on different contigs, whereas *ctxAB*, *zot*, and *ace* of 89/104 isolates were on the same contig.

All 104 genomes contained 19 *Vibrio* pathogenicity island genes, 18 genes on 2 *Vibrio* seventh pandemic islands, and 19 type VI secretion system–related genes. All but 1 genome contained the intact repeats-in-toxin gene cluster. (Appendix 1 Table 2). We found *Vibrio* pathogenicity island genes on the same contigs in 99/104 genomes, *Vibrio* seventh pandemic island I genes on the same contigs in 103/104 genomes, and *Vibrio* seventh pandemic island II genes on the same contigs in 104/104 genomes.

Because a strain may contain multiple copies of the CTX phage (*24*), we used CNVnator to estimate the number of copies of the *ctxB* gene in the 104 isolates by mapping reads to *V. cholerae* seventh pandemic reference genome N16961. The *ctxB* gene copies differed in the 3 clusters; on average, C2 had 4.4 copies/isolate, C3 had 1.2 copies/isolate, and C1 had 1.3 copies/isolate. A total of 68 isolates carried multiple copies of *ctxB* (range 4–22 copies) (Appendix 1 Table 2), whereas 38 isolates carried only 1 copy of *ctxB*. Most C2 isolates (81.4% [48/59]) carried >2 copies (average 5 copies) of *ctxB*. In contrast, only 3 isolates (10.7% [3/28]) in C3 carried >1 copy (average 2.6 copies), and 6 isolates (54.5% [6/11]) in C1 carried 2 copies. Six outlier isolates carried multiple copies of *ctxB* (range 2–22 copies). All 101 *ctxB*–positive isolates contained *ctxB* genotype 3.

### Antimicrobial-Resistance Genes and Resistance Mutations of the O139 Isolates

We found the chromosomally encoded resistance genes *varG* and *catB9* in all isolates. *floR*, *dfr18*, *sul2*, *aph(3′′)-lb*, and *aph(**6**)-ld* were in most of the isolates, including international isolates, and 8 AMR genes detected only in cluster 3 were present at low frequencies (3.13%–15.63%) (Table). We found *bla*_TEM-1_, *catA2*, *aac(**3**)-lld*, *aadA2*, *aph(3′)-la*, *mph(E)*, *msr(E)*, *sul*, *dfrA12*, *tet(M)*, and *tet(Y)* only in isolates from China, including Shanghai isolates, whereas *bla*_CMY-2_, *bla*_OXA-1_, *catB3*, *aac(6′)-lb-cr5*, *aadA3*, *ere(A)*, *mph(A)*, *mph(F)*, *qnrA1*, *qnrA7*, *aar-3*, *dfrA27*, *dfrA32*, *tet(A)*, *and tet(D)* were in Zhejiang isolates only (Appendix 1 Table 4). C1 carried only AMR genes common to all isolates, *aph(3′)-Ia and sul1* were common to C2 and C3, *aac(**3**)II*, *aadA2*, *tet(D)*, *mph(E)*, *msr(E)*, *bla*_TEM-1_, and *catA2* were more common in C2, and *tet(M)*, *mph(A)*, and *dfrA12* were more common in C3.

**Table:** Antimicrobial resistance gene profiles in 3 clusters of *Vibrio cholerae* O139 isolates from Zhejiang Province, China, 1994–2018, and in groups of isolates from outside of China

Gene	Cluster or group, no. (%)				
	Cluster 1, n = 11	Cluster 2, n = 63	Cluster 3, n = 32	Lineage 1 non-China, n = 15	Other* non-China, n = 33
*aph (3′′)-Ib*	11 (100)	59 (93.65)	31 (96.88)	6 (40)	33 (100)
*aph (6)-Id*	11 (100)	59 (93.65)	31 (96.88)	6 (40)	33 (100)
*dfrA18*	11 (100)	35 (55.56)	29 (90.63)	1 (6.67)	33 (100)
*sul2*	11 (100)	63 (100)	32 (100)	5 (33.33)	33 (100)
*varG*†	11 (100)	63 (100)	32 (100)	15 (100)	33 (100)
*catB9*†	11 (100)	63 (100)	32 (100)	15 (100)	33 (100)
*floR*	11 (100)	59 (93.65)	31 (96.88)	6 (40)	33 (100)
*bla*_TEM-1_	0	61 (96.83)	0	0	0
*aph(3′)-Ia*	0	57 (90.48)	23 (71.88)	0	0
*aadA2*	0	56 (88.89)	5 (15.63)	0	0
*catA2*	0	56 (88.89)	4 (12.50)	0	0
*tet(D)*	0	55 (87.30	9 (28.13)	0	0
*sul1*	0	54 (85.71)	26 (81.25)	0	0
*aac(3)-IId*	0	53 (84.13)	0	0	0
*mph(E)*	0	52 (82.54)	2 (6.25)	0	0
*msr(E)*	0	52 (82.54)	2 (6.25)	0	0
*mph(A)*	0	0	22 (68.75)	0	0
*tet(M)*	0	0	21 (65.63)	0	0
*dfrA12*	0	2 (3.17)	21 (65.63)	0	0
*tet (Y)*	0	5 (7.94)	2 (6.25)	0	0
*aac(6')-Ib-cr5*	0	0	1 (3.13)	0	0
*aadA16*	0	0	4 (12.50)	0	0
*aadA3*	0	0	4 (12.50)	0	0
*arr-3*	0	0	4 (12.50)	0	0
*bla*_CMY-2_	0	0	1 (3.13)	0	0
*bla*_OXA-1_	0	0	1 (3.13)	0	0
*catB3*	0	0	1 (3.13)	0	0
*dfrA27*	0	0	4 (12.50)	0	0
*dfrA32*	0	0	1 (3.13)	0	0
*ere(A)*	0	0	1 (3.13)	0	0
*mph(F)*	0	0	1 (3.13)	0	0
*qnrA1*	0	0	3 (9.38)	0	0
*qnrA7*	0	0	1 (3.13)	0	0
*tet(A)*	0	0	4 (12.50)	0	0

*Isolates not grouped in lineage 1 and clusters 1–3 and not isolated in China (Appendix 1 Table 4). †Genes that have not been associated with phenotypic resistance, determined on the basis of published data up to now.

We also searched these isolates for quinolone-resistance mutations. Seventy-four isolates (74/104 [71.2%]) harbored mutations Ser85Leu in *parC* and Ser83Ile in *gyrA*. Ten isolates had a mutation in Asp87 of *gyrA*, of which 6 had Asp87Tyr, 3 had Asp87Gly, and 1 had Asp87Asn.

### Association of Plasmids and Integrative and Conjugative Elements with AMR Genes

We analyzed the integrative and conjugative elements (ICEs) carried by our isolates and compared them with the 2 known ICE variants in O139 (SXT^MO10^ and ICE*Vch*Ind4) (*25*). SNP and phylogenetic analyses found that all O139 ICEs were closely related (difference of 0–13 SNPs) (Appendix 2 Figure 5). Our ICEs differed from ICEVchInd4 by 0–7 SNPs and from SXT^MO10^ by 10–13 SNPs. However, most of our isolates carried the 4 SXT^MO10^ genes, including *dfr*A18 that are absent in ICE*Vch*Ind4. The AMR genes present in our isolates, *dfr*A18, *floR*, *aph(3′′)-Ib*, *aph(**6**)-Id*, and *sul2*, were probably carried by the ICE.

Eighty-three isolates carried an IncA/C plasmid. We found 2 IncA/C subtypes (IncA/C2_1_JN157804 type, belonging to plasmid pNDM-KN-lineage, and IncA/C_1_FJ705807 type, belonging to pRA1-lineage) (Appendix 1 Table 4). None of the C1 isolates contained an IncA/C plasmid. All except 2 C2 isolates contained an IncA/C2 plasmid (Appendix 1 Table 4). k-mer alignment analysis indicated that these C2 isolates carried a plasmid nearly identical to the known *V. cholerae* plasmid pVC1447 of 160 kb (*9*). pVC1447 is known to carry *aadA*, *sul1*, *tetD*, *bla*_TEM_, *catA2*, *mph(E)*, *tet(R)*, *mel*, *qacEdelta1*, and *folP* genes (*9*). The last 4 AMR genes were not found in any of our C2 isolates. Most C2 isolates carried *aadA2*, *sul1*, *tetD*, *catA2*, *mph(E)*, *msr(E)*, and *bla*_TEM_. Seven C2 isolates lost >1 of the AMR genes. Isolate V29 and V30 lost *sul1*, *tetD*, *mph(E)*, and *msr(E)* genes. The 2 C2 isolates without the pVC1447-like plasmid did not contain any of the pVC1447 AMR genes. All except 4 C3 isolates carried an IncA/C plasmid; 17 had the IncA/C_1_FJ705807 replicon type, and 5 had the IncA/C2_1_JN157804 replicon type. We further determined that the IncA/C_1_FJ705807 type plasmid is a novel plasmid that was most closely related to *Aeromonas veronii* plasmid p158496 (*26*), whereas the IncA/C2_1_JN157804 type plasmid was most similar to *V. cholerae* O139 pVC211 (GenBank accession no. KY399978.1). The p158496-like plasmid in the 17 C3 isolates shared an average nucleotide identity of 97.7% and length coverage of 82.89% with the 158 kb p158496 and probably carried *aadA2*, *tet(D)*, *tet(M)*, *mph(A)*, *dfrA12*, and *sul1* genes. However, more than half of these C3 isolates lost the *tet(D)* gene*.* Two outlier isolates (V17 and V18) also carried the p158496-like plasmid. The pVC211-like plasmid in the 5 C3 isolates shared an average nucleotide identity of 99.09% and length coverage of 92.46% with the 148 kb pVC211 plasmid and probably carried *aadA16*, *tet(A)*, *mph(A)*, *dfrA27*, *qnrA1*, and *arr-3* resistance genes. Some isolates had further loss and gain of AMR genes.

## Discussion

The first *V. cholerae* O139 isolate in Zhejiang Province was reported in September 1994, which was 16 months after the first O139 case reported in China (*6*). Phylogenetic analysis grouped Zhejiang isolates into 2 independent lineages (L1 and L2) and 3 clusters (C1, C2, and C3). The origin of C1 was probably India and the origin of L2 (C2 and C3) was probably Thailand. However, considerable uncertainty exists, as it does with L1, the sister clade of C1, which contained both India and Bangladesh isolates. Similarly, L2, which contained C2 and C3 of isolates from China only, shared a most recent common ancestor with isolates from India, Bangladesh, and Thailand. More isolates from the other countries in Asia would be required to resolve the origins of the clusters in China.

Other isolates in China also fell into the 3 Zhejiang clusters, suggesting that these clusters were circulating across China. However, because the publicly available O139 genomes from other parts of China contained no location metadata, we cannot infer whether O139 reached Zhejiang first and then spread to other parts of China or vice versa.

All isolates in this study were *ctxB* genotype 3. However, a study of isolates from south China found that a small proportion of *ctxB* genotypes 1 and 5 in isolates from the 1990s, although >90% of the isolates were *ctxB* genotype 3 (*7*). Most C2 isolates carried multiple copies of *ctxB*, suggesting that the cluster carried multiple copies of the CTX phage. The number of CTX carried by O139 may vary (*24*). In our study, we observed that the variation in the number of CTX carried occurred along lineages. The higher number of *ctx* copies might lead to greater toxin production, potentially affecting disease outcomes.

On the basis of the presence of AMR genes and resistant mutations, we determined that the evolution of resistance to antimicrobials changed substantially over time. Tetracycline resistance genes *tet*(M) and *tet*(Y) were present only in isolates in China, and *tet*(A) and *tet*(D) were only present in Zhejiang isolates. *tet*(M) was found in 65.6% of C3 isolates; some C3 isolates carried both *tet*(D) and *tet*(M). Previous studies found that O139 isolates from 1991–2013 in Thailand and from 1997 in India were susceptible to tetracycline (*27*,*28*), suggesting that the earlier O139 isolates in Asia did not carry the *tet* genes. Because tetracyclines were overused in China (*29*), it is not surprising that C2 and C3 isolates acquired tetracycline-resistance genes, and these events probably occurred in China.

*mph*(A) was present in 68.8% of C3 isolates only. *mph*(A) conferring azithromycin resistance is plasmid-borne and rarely found in *V. cholerae* (*30*). Azithromycin was first used in clinical treatment in 1988 (*31*). In our study, *mph*(A) was first identified in an isolate in 1998, only 10 years after azithromycin was first used for treatment. The high percentage of *mph*(A) in O139 C3 isolates in this study is concerning. However, because cholera was relatively infrequent in China, the acquisition of such resistance may not be attributable to selection pressure from clinical antimicrobial treatment.

C2 and C3 shared 2 nonsynonymous mutations, 1 each in the genes encoding for penicillin-binding protein 2 and a lytic murein transglycosylase with affinity to β-lactam antibiotic resistance (*32*). These genomic changes were previously reported in Shanghai O139 isolates and were attributed to the increasing usage of β-lactam antibiotics (*10*). These mutations were present only in isolates originating in China in L2 and may have evolved in China.

Plasmid analysis found that C2 and C3 isolates acquired different IncA/C plasmids. C2 carried a known plasmid (pVC1447), whereas C3 isolates acquired 2 different IncA/C plasmids, *A. veronii* plasmid p158496-like and *V. cholerae* pVC211-like. These plasmids were probably the carriers of the new AMR genes in different clusters and contributed to the differences of AMR gene profiles between clusters.

Although O139 spread to China in 1993 (*6*), our earliest isolates in Zhejiang were from 1994 and did not belong to any of the 3 clusters. Five more unclustered isolates were from 1996–1998, all of which belong to L2. Therefore, in early years of the O139 epidemic, multiple independent introductions of O139 cholera to Zhejiang directly from other countries or indirectly from other parts of China had probably occurred. However, the 3 clusters flourished at different times were successively replaced during 1994–2018. C1 was found in 1996 and persisted until 2000, C2 during 1998–2008, and C3 during 1998–2018. The earliest isolate from both C2 and C3 were 1998, suggesting that C2 and C3 were imported to China at similar times or a single importation of the most recent common ancestor of C2 and C3 had occurred from which the 2 clusters diverged in China. C2 became a dominant population in Zhejiang during 2000–2008 and then C3 took over from 2009, replacing the other clusters. Therefore, Zhejiang experienced O139 cholera in 3 waves caused by 3 clusters, each lasting up to a decade. 

The epidemiologic pattern uncovered raises many interesting questions, most notably regarding what advantage did subsequent clusters have over their predecessor. C2 and C3 carried more resistance genes than C1. Although we have no AMR phenotypic data, difference in their AMR gene profiles suggests that AMR may have been the driver that caused C1 to be replaced by C2 and C3. C2 overall carried more copies of *ctxB*, suggesting that it may produce more CTX toxin than C3. Nearly 50% of the isolates from other parts of China from the unpublished genomes belonged to C2, suggesting that C2 was quite prevalent and more successful than C3. The increased number of copies of *ctxB* probably contributed to its success in replacing C1. However, this explanation does not account for why C2 was subsequently replaced by C3 in Zhejiang. Again, C3 acquired resistance to several additional AMR genes that may explain its fitness advantage over C2, given that *tet*(M) and *mph*(A) were only detected in C3 and *dfrA12* was mainly present in C3. In addition, the AMR genes present at low frequency in different C3 isolates may have also collectively contributed to C3’s fitness. However, 63% of C2 isolates simultaneously carried *blaTEM1*, *catA2*, *aac(**3**)-lld*, *aadA2*, *aph(3′)-la*, *sul1*, *mph(E)*, *and msr(E)* genes, a pattern not present in other clusters. 

The persistence of each cluster for many years in Zhejiang is also intriguing. The clusters possibly were circulating in other parts of China and spread to Zhejiang. Most of the other isolates in China fell into C2, and Shanghai isolates were shown to be ancestral to some Zhejiang isolates within C2 and C3 (Appendix 2 Figure 2), supporting this hypothesis. Isolates may have also been continuously imported from other countries. However, we have no isolates from other countries of corresponding years to examine this hypothesis. Another possibility is that O139 has spread to the environment in Zhejiang, where it has established itself as a local reservoir. However, our extensive sampling of river waters over 2 years in 2 cities in Zhejiang only found non-O1/non-O139 isolates (*33*), although the sampling done in that study had only 2 years overlap with the isolation years of O139 isolates from those cities. Thus, it is less likely that these O139 cases were from local environmental reservoirs. A recent study of cholera in Africa also found repeated importation rather than local environmental reservoirs as the source of the seventh pandemic cholera during cholera resurgence over a 40-year period (*11*).

This study describes the possible origin, evolution, and spread of O139 cholera in a single province, Zhejiang. Further studies are required to expand this analysis to the national level. Most *V. cholerae* O139 isolates in Zhejiang grouped into 3 major clusters, which were probably derived from multiple independent importation events directly or indirectly from other countries in Asia and prevailed over the period 1994–2018, with one cluster replacing another sequentially. Variations in AMR gene content or resistance mutations suggest that acquisition of AMR probably has played a role in the succession of the *V. cholerae* O139 clusters in Zhejiang.

Appendix 1 Additional data for study of genomic epidemiology of *Vibrio cholerae* O139, Zhejiang Province, China, 1994–2018.

Appendix 2 Additional information about study of genomic epidemiology of *Vibrio cholerae* O139, Zhejiang Province, China, 1994–2018.
