# Supplementary material for: Genomic Epidemiology of Vibrio cholerae O139, Zhejiang Province, China, 1994–2018
Source: Emerg Infect Dis. 2022 Nov;28(11):2253–60. doi: 10.3201/eid2811.212066 (PMC9622232; doi:10.3201/eid2811.212066)
Supplement: Appendix 2 — Additional information about study of genomic epidemiology of Vibrio cholerae O139, Zhejiang Province, China, 1994–2018. [file 21-2066-Techapp-s2.pdf]

Article DOI: https://doi.org/10.3201/eid2811.212066

Genomic Epidemiology of *Vibrio cholerae* O139, Zhejiang Province, China, 1994–2018

Appendix 2

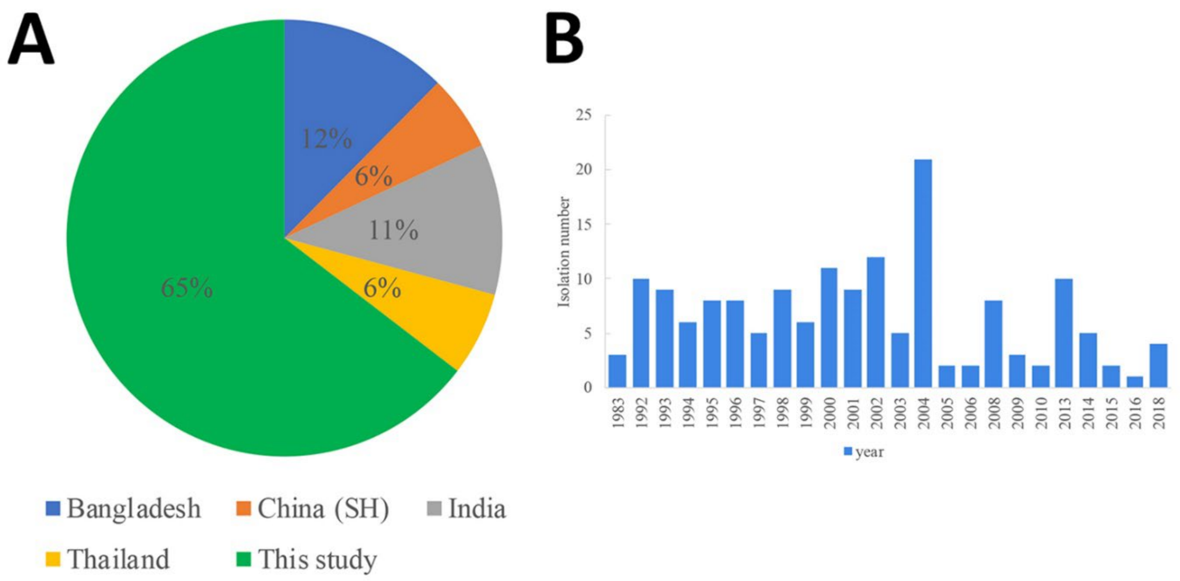

Appendix 2 Figure 1. Distribution of 161 isolates in this study. Pie chart shows the distribution of the sources of the isolates. Bar chart shows the distribution of years of isolation of the isolates.

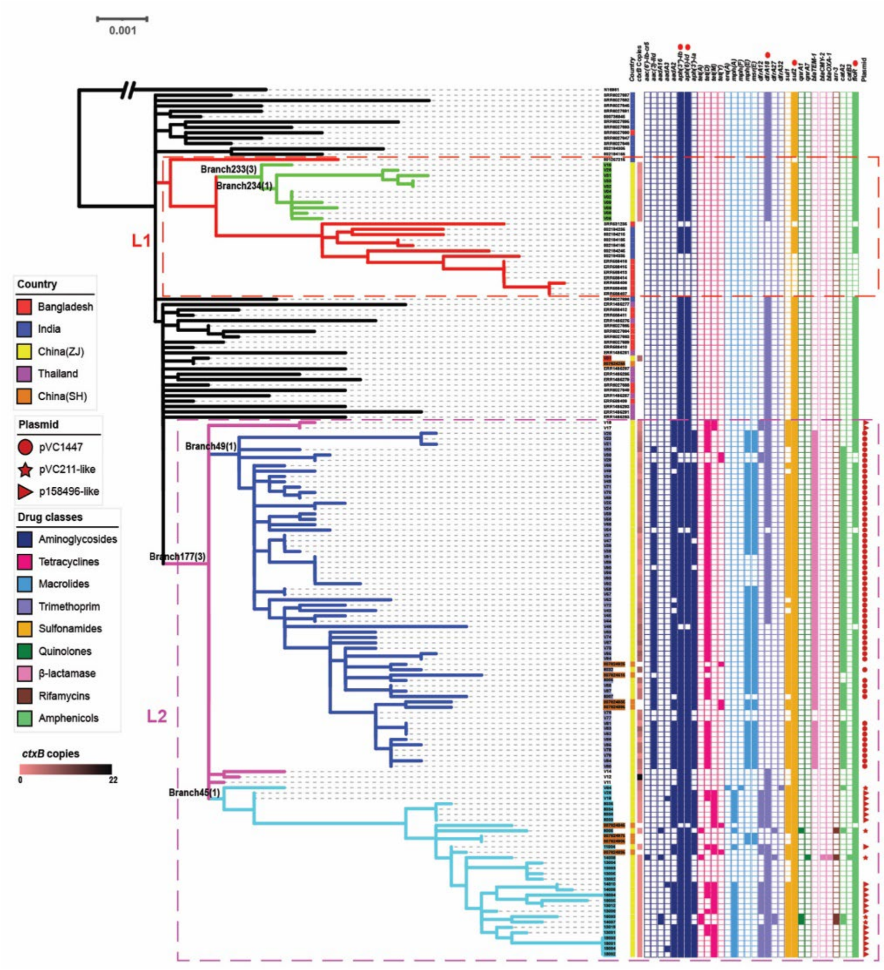

Appendix 2 Figure 2. Maximum likelihood tree of the *V. cholerae* O139 (ST69)161 isolates and their resistance profiles. The tree was rooted using the 7th pandemic O1 strain N16961 as an outgroup. Lineage 1 (L1) and Lineage 2 (L2) were demarcated with red dash line and pink dash line boxes. Branches for different clusters were colored for easy visualization. The isolates with brown labels were from China (Shanghai). The isolate marked with a red star was the first O139 *V. cholerae* in Zhejiang (V01). Key branches were marked with a branch number followed in brackets of the number of SNPs that supported the branch. On the right side of the tree were metadata and genetic information as shown on the heading of each column. *ctxB* gene copies were marked as gradient of pink from zero copy to 22 copies while no color meant no data available. Antibiotic resistance genes were grouped by drug classes with color legend as shown, although presence of a resistance gene or mutation may not confer resistance to a given drug phenotypically. Gene *varG* and *catB9* were present in all the genomes but not associated to phenotypic resistance based on published data up to now therefore they were removed from the figure. The AMR genes associated with SXT/ICEVchInd4 was labeled with red dots on top of the gene names. Filled and empty squares represent gene presence and absence respectively. Red circle represents plasmid pVC1447, red star represents pVC211-like plasmid and red triangle represents p158496-like plasmid.

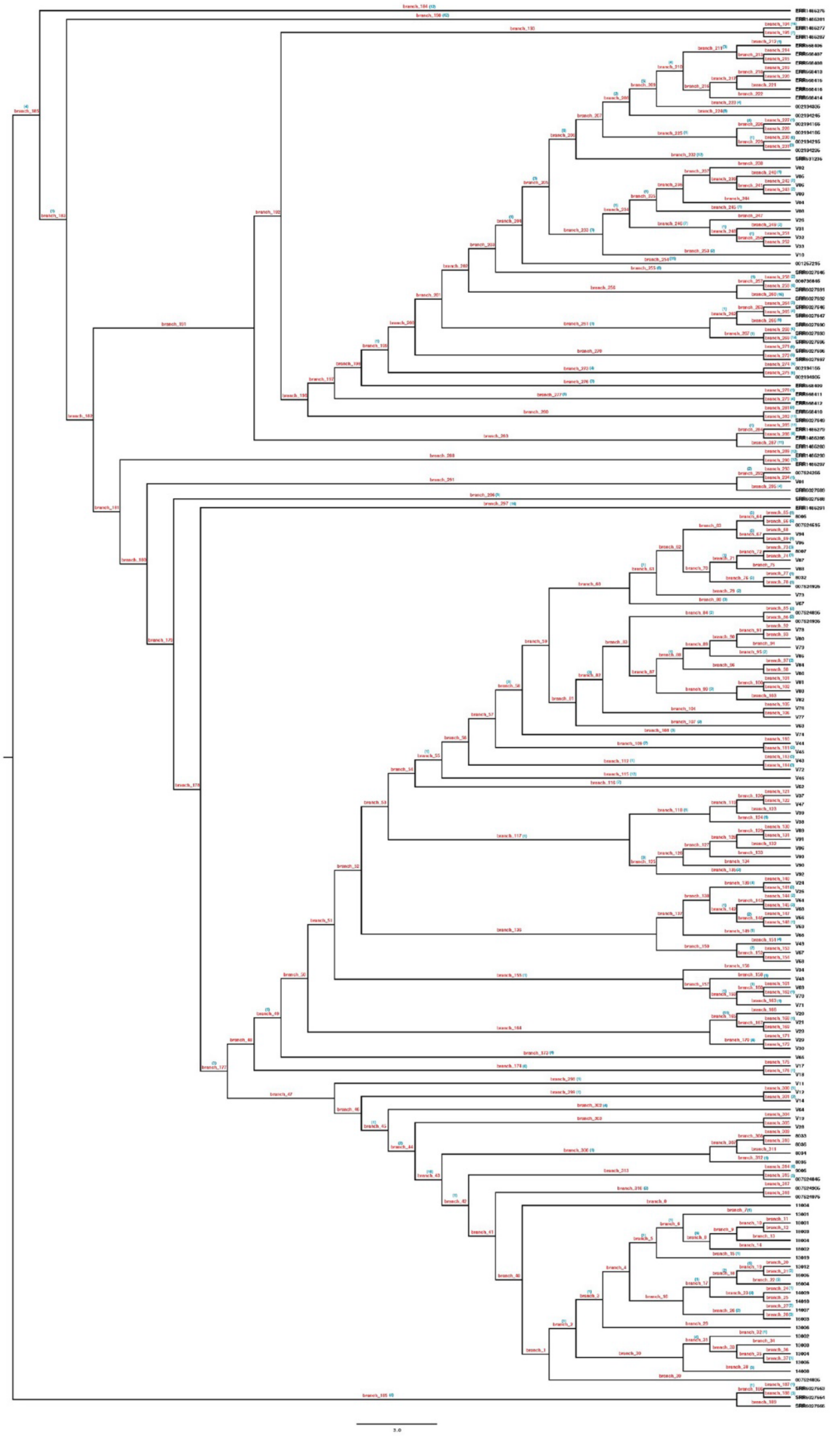

Appendix 2 Figure 3. Branch identifications and SNP numbers on each branch of 161 genomes. Branch IDs were marked on each branch and the SNP numbers were in blue brackets.

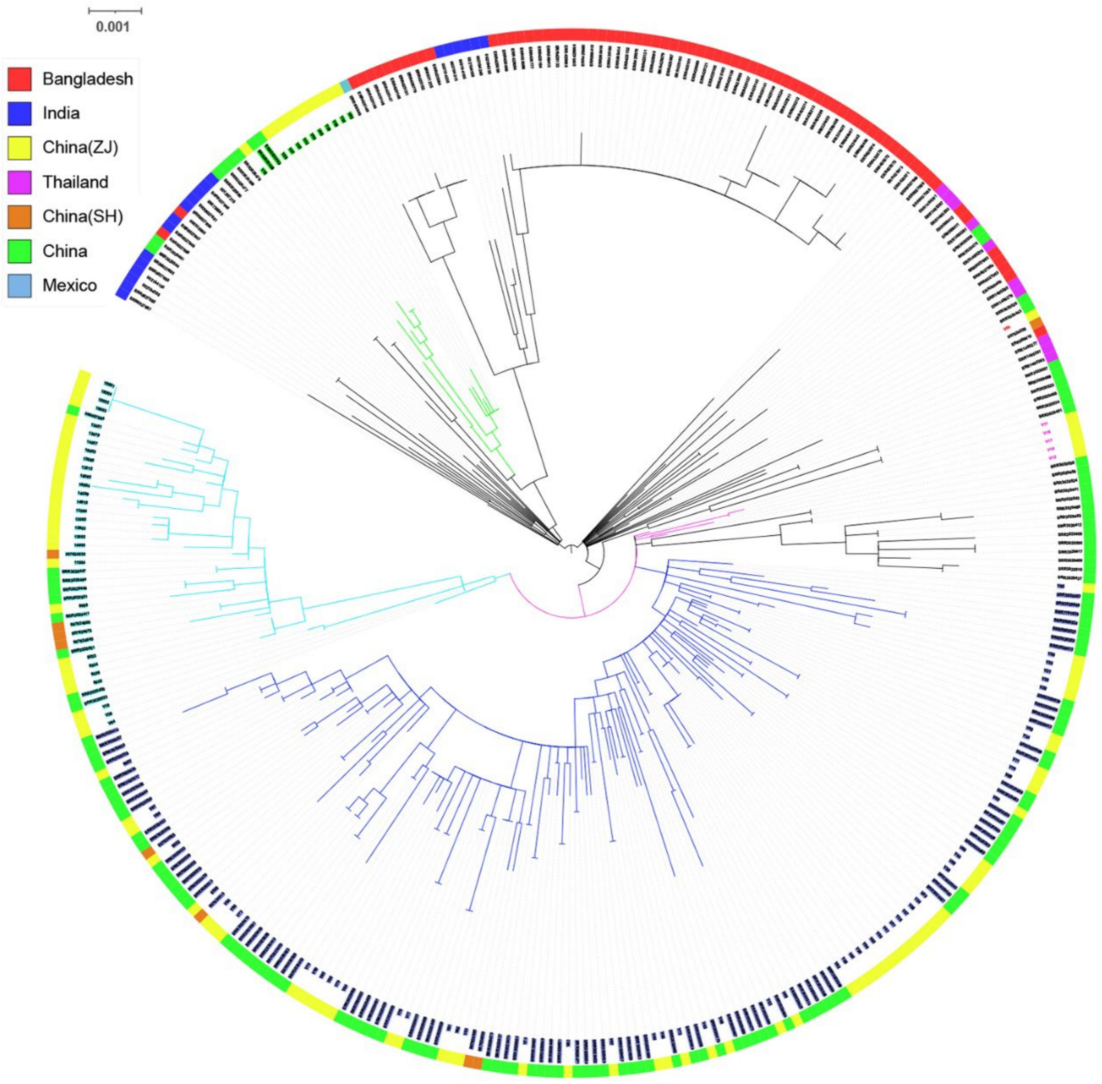

Appendix 2 Figure 4. Phylogenetic tree of 349 genomes. 133 genomes from other parts of China without location metadata and another 55 genomes in an independent lineage were included in this tree to infer the phylogenetic relationship with the data in this study. All the lime branches were Cluster 1(C1), blue branches were Cluster 2 (C2) and aqua branches were Cluster 3 (C3). The pink branches were five isolates from Zhejiang but unclustered. The support from 1000 bootstrap replicates is shown on each branch.

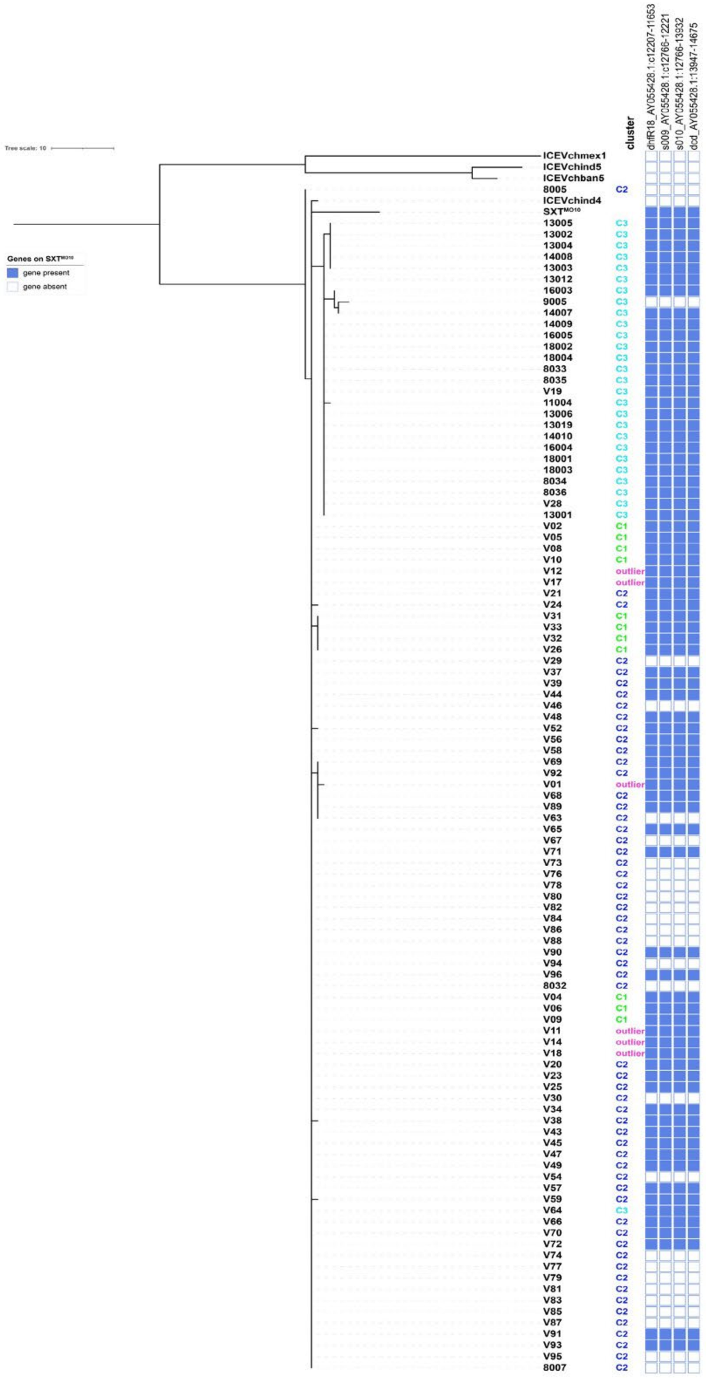

Appendix 2 Figure 5. Phylogenetic relationship of integrating conjugative elements (ICEs) in ZJ isolates SNPs were called against the SXT elements (AY055428.1) in a *Vibrio cholerae* strain MO10 and used to construct a parsimony tree including three ICEs in *V. cholerae* O1 (ICEVchind5, ICEVchmex1 and ICEVchban5) and two ICEs in V. cholerae O139 (ICEVchind4 and SXT^{MO10}). Four genes located on a unique fragment (AY055428.1:11531–14859) in SXT^{MO10} were screened using BlastP and marked present or absent in blue squares. Isolate names and their clusters were as shown to the right of the tree.
